# Proteomic Profiling of Rabbit Embryonic Stem Cells Derived from Parthenotes and Fertilized Embryos

**DOI:** 10.1371/journal.pone.0067772

**Published:** 2013-07-04

**Authors:** Payungsuk Intawicha, Shih-Han Wang, Ya-Chen Hsieh, Neng-Wen Lo, Kun-Hsiung Lee, San-Yuan Huang, Jyh-Cherng Ju

**Affiliations:** 1 Department of Animal Science, National Chung Hsing University, Taichung, Taiwan, ROC; 2 Department of Animal Science and Biotechnology, Tunghai University, Taichung, Taiwan, ROC; 3 Division of Biotechnology, Animal Technology Institute Taiwan, Miaoli, Taiwan, ROC; 4 Agricultural Biotechnology Center, National Chung Hsing University, Taiwan, ROC; 5 Center of Nanoscience and Nanotechnology, National Chung Hsing University, Taiwan, ROC; Wellcome Trust Centre for Stem Cell Research, United Kingdom

## Abstract

Rabbit embryonic stem (rES) cells can be derived from various sources of embryos. However, understanding of the gene expression profile, which distincts embryonic stem (ES) cells from other cell types, is still extremely limited. In this study, we compared the protein profiles of three independent lines of rabbit cells, i.e., fibroblasts, fertilized embryo-derived stem (f-rES) cells, and parthenote-derived ES (p-rES) cells. Proteomic analyses were performed using two-dimensional gel electrophoresis (2-DE) and mass spectrometry. Collectively, the expression levels of 100 out of 284 protein spots differed significantly among these three cell types (*p<0.05*). Of those differentially expressed spots, 91% were identified in the protein database and represented 63 distinct proteins. Proteins with known identities are mainly localized in the cytoplasmic compartments (48%), nucleus (14%), and cytoskeletal machineries (13%). These proteins were majorly involved in biological functions of energy and metabolic pathways (25%), cell growth and maintenance (25%), signal transduction (14%), and protein metabolisms (10%). When protein expression levels among cell types were compared, six proteins associated with a variety of cellular activities, including structural constituents of the cytoskeleton (tubulins), structural molecule (KRT8), catalytic molecules (α-enolase), receptor complex scaffold (14-3-3 protein sigma), microfilament motor proteins (Myosin-9), and heat shock protein (HSP60), were found highly expressed in p-rES cells. Two proteins related to HSP activity and structural constituent of cytoskeleton in f-rES cells, and one structural molecule activity protein in fibroblasts showed significantly higher expression levels (*p<0.05*). Marker protein expressions in f-rES and p-rES cells were further confirmed by Western blotting and immunocytochemical staining. This study demonstrated unique proteomic profiles of the three rabbit cell types and revealed some novel proteins differentially expressed between f-rES and p-rES cells. These analyses provide insights into rES cell biology and would invite more in-depth studies toward rES cell applications.

## Introduction

Rabbit embryonic stem (rES) cells are pluripotent cells derived from the blastocyst stage embryos [1]–[2]. Recently, more newly established rES cells are derived from fertilized embryos (f-rES) [3]–[9]. Parthenogenetically activated (PA) oocytes or embryos are subjected to artificial stimuli to initiate embryonic development without fertilization process or incorporation of sperm chromosomes. These parthenotes possess chromosomes entirely of the maternal origin and fail to develop to term due to a lack of paternal gene expressions or normal genomic imprinting [10]–[11]. Similar to f-rES cells, parthenote-derived rES (p-rES) cells can continuously proliferate *in vitro,* retain self-renewal capacity without differentiation [3]–[12], and also differentiate into cell lineages of the three germ layers both *in vitro* and *in vivo*. They expressed the same set of pluripotency marker genes *(Oct4, Nanog,* and *Sox2*), alkaline phosphatase (AP), and proteins such as cell surface markers including stage specific embryonic antigen-4 (SSEA-4), keratin sulfate antigens TRA-1-60, and TRA-1-81 as well as octamer-binding transcription factor (Oct4) [3]–[4], [12], so did the f-rES cells. However, there are fundamental differences between f-rES and p-rES cells in that p-rES cells are generated from homozygous embryos consisting of only haploid female genome and lack of the expression of paternally imprinted genes such as *Snrpn* and *Igf2* [3], [12]. With these properties, p-rES cells have been proposed and proved to be useful in ameliorating or completely eliminating the risk of immunological rejection after cell transplantation [13].

Recently, proteomic analyses have been performed to monitor the global protein expression and post-translational modifications in mouse ES (mES) cells [14]–[16], and to determine the protein expression profiles in mouse [17]–[18], monkey [19], and human [20]–[21] ES cells undergoing chemically induced differentiation. So far, no authentic germline transmissible rES cell lines were reported and the molecular mechanisms superimposing distinct characteristics onto the f-rES and p-rES cells are largely unknown. Apparently, understanding of the differentially expressed protein profiles between the p-rES and f-rES cells becomes of importance for further in-depth studies toward rES cell authenticity and cell replacement therapies. In the present study, proteomic and bioinformatic analyses on the three rabbit cell types (fibroblasts, f-rES cells, and p-rES cells) were performed to unravel the distinctive protein expression profiles among them.

## Materials and Methods

### Animal Use and Reagents

The care and use of all animals for recovering embryos were complied with the guidelines and was approved by the Institutional Animal Care and Use Committee (IACUC) of National Chung Hsing University, Taiwan, ROC (IACUC Permit NO. 96-72). Chemicals and reagents used were mainly purchased from Sigma-Aldrich Co. unless otherwise mentioned.

### Culture of rES Cells and Fibroblasts

**Figure 1:**
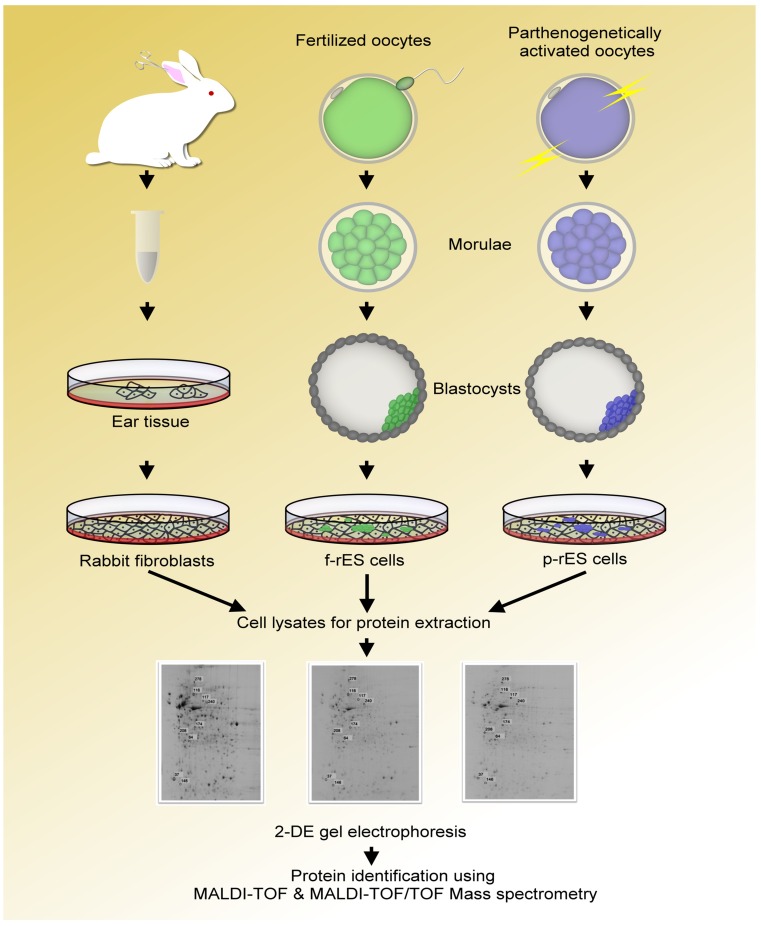
The schematic diagram shows the experimental procedures of the 2-DE-based proteomic analysis in this study. Three rabbit cell types (fibroblast, f-rES, and p-rES cells) were used for soluble protein extraction and analysis. The expression levels of protein spots were compared among all cell types.

The schematic diagram of the experimental procedures for the proteomic analysis in this study is shown in Fig. 1. One representative f-rES cell line (R4) derived from the blastocyst of fertilized embryos [8] and one p-rES cell line (A2) from parthenotes [12] were used for 2-DE analysis. The f-rES and p-rES cell lines were cultured in 81.5% D-MEM/F-12 (Cat. No. 12400-024, Gibco Products International, Grand Island, NY, USA) supplemented with 15% FCS (Cat. No. 10437-028, Gibco Products International), 2 mM L-glutamine (Cat. No. G8540-25, Sigma-Aldrich, St. Louis, MO, USA), 1% nonessential amino acids (Cat. No. M7145, Sigma-Aldrich), 0.1 mM β-mercaptoethanol (Cat. No. M7522, Sigma-Aldrich), 1,000 U/mL recombinant mouse leukemia inhibitory factor (LIF; Cat. No. ESG1107, Chemicon, Temecula, CA, USA) and/or 4 ng/mL human recombinant basic fibroblast growth factor (bFGF; Cat. No. CYT-218, Prospec, Rehovot, Israel) using MEF as feeder cells, described in the previous study [8]. The ES cells were passaged every 4 days and the medium was changed every other day. Rabbit fibroblasts cultured in 90% DMEM (Cat. No. D7777, Sigma-Aldrich) with 10% FCS (Cat. No. 10437-028, Gibco Products International) served as a comparative control of the differentiated cell population. All cells were maintained in a humidified incubator (38°C) containing 5% CO_2_ in air, and protein samples of rES cells at passage 15 and fibroblasts were prepared as described below.

### Reverse Transcription-polymerase Chain Reaction (RT-PCR)

Rabbit ES cell colonies were lifted from feeders with dispase (1 mg/mL; Gibco17105041) at 37°C and fibroblasts were subject to trypsinization. The procedures for RNA extraction were performed as previously described [8], [12]. Total RNAs of rabbit fibroblasts, f-rES, and p-rES cells were extracted using a total RNA extraction kit (Geneaid RT050, Taipei, Taiwan, R.O.C.). Primer sequences including sense and antisense, annealing temperatures, and expected sizes of amplicons are used following: *Snrpn*: 5′-TGAGACGGACTACAGAGCAG-3′, 5′-GGTATGATGGCAGGTTCTCC-3′ (59°C, 279 bp); *Igf2*∶5′-GCTTCTACTTCAGCAGGC-3, 5′- GTGTCATATTGGAAGAACTTG-3′ (54°C, 207 bp); *GAPDH*: 5′-GGAGCCAAACGGGTCATCATCTC-3′, 5′-GAGGGGCCATCCACAGTCTTCT-3′ (62°C, 233 bp).

### Sample Preparation for Proteomic Analysis

To prevent contamination of feeder cells with rES cells, rES cell colonies were lifted from feeders by treatment with dispase (1 mg/mL; Gibco17105041) at 37°C for 1–2 min for gentle cracking, and then rES cell culture medium was added to stop the enzymatic reaction. The colonies were collected into a 15-mL tube and stood for 3 min to settle and separate rES colonies from feeder cells. Old medium in the tube was replaced with fresh medium (10 mL) and then the tube was again left still for 3 min to set down rES cell colonies. The same procedures were repeated for three times to remove feeder cells from rES cells for analyses. For proteomic analysis, cultured rabbit fibroblasts and rES cells were washed with DPBS (Cat. No 21600-051, Gibco Products International) then trypsinized to single cells and centrifuged at 80×g. The cell pellets were frozen in liquid nitrogen and stored at -80°C for further analysis. Cell samples (>10^6^ cells per sample) were lysed in lysis buffer (9.5 M urea, 65 mM DTT, 2% Ampholyte pH 3–10, and 2% NP-40) and then frozen at -80°C for 20 min. After thawing and centrifugation at 19,000×g for 5 min, the supernatant was collected. Protein concentrations were determined by the Ettan 2-D Quant kit (GE Healthcare, Bio-Science AB, Uppsala, Sweden) using BSA as the standard. A total of 1,000 µg soluble proteins were subject to trichloroacetic acid (TCA) precipitation before analyses. Briefly, equal volume of 20% TCA was added to the sample and then incubated on ice for 1 h (vortexed every 15 min). The sample was then centrifuged and the supernatant was discarded. The pellets were washed twice with two volume of 90% ice-cold acetone and centrifuged at 19,000×g at 4°C for 10 min. The pellet was then lyophilized and dissolved in lysis buffer for protein analysis.

### Protein Analysis by 2-DE

The 2-DE procedure was based on Görg *et al*. [22] with some modifications [23]. Briefly, 1,000 µg TCA-precipitated protein in 175 µL lysis buffer was mixed with an equal volume of rehydration buffer (8 M urea, 2% CHAPS, and 0.5% (v:v) Pharmalyte (pH 3 to pH 10), and then subject to IEF using 18-cm immobilized pH gradient (pH 3–10) strips on an Ettan IPGphor 3 (GE Healthcare, Bio-Science AB, Uppsala, Sweden) at 20°C. The strip was rehydrated at 30 V for 12 h and further focused for 64,000 voltage-hour. The focused strips were equilibrated first in equilibration solution (50 mM Tris-Cl (pH 6.8), 6 M urea, 30% (w/v) glycerol, 2% (w/v) SDS, and 0.002% (w/v) bromophenol blue) containing 100 mM DTT for 20 min, and then the strips were further equilibrated in equilibration solution containing 150 mM iodoacetamide for 20 min. After equilibration, proteins were separated by 12.5% SDS-PAGE using the Daltsix Vertical electrophoresis system (GE Healthcare, Bio-Science AB). The separation was run at 15°C with a condition of 2.5 Watts per gel for 25 min followed by 9 Watts per gel until the dye front reached the bottom of the gel (typically 7–7.5 h). The molecular weight (*Mr*) standards were purchased from Fermentas (#SM0661 Unstained Protein Ladder, Vilnius, Lithuania) containing synthesized peptides with the molecular weights ranging from 10 to 200 kDa.

### Staining and Imaging of the 2-DE Gels

After protein separation, gels were stained with colloidal Coomassie blue (Serva Electrophoresis GmbH, Heidelberg, Germany) for at least 14 h [24]. Following staining, the gel was neutralized with 0.1 M Tris/phosphoric acid (pH 6.5) for 1 to 3 min, and then destained with 25% methanol. After destaining, gels were scanned at a resolution of 300 dpi with a gel scanner (Image Scanner III, GE Healthcare, Bio-Science AB) and saved as TIFF image files for further analysis.

### Analyses of Protein Expression Levels in Different Cell Types

For each individual cell line, three 2-DE profiles were used for image analyses. Protein spots on the 2-DE gels were detected and analyzed using the Melanie 7 software package (GeneBio, Geneva, Switzerland). All the spots detected on the profiles were then grouped and quantified for spot volume by the software. Relative volumes of each spots were calculated to represent the expression levels. Ratios of the volume (RVol) of each spot to total volume of all quantified spots on each gel were generated by the software to correct variations in gel staining [25]. The RVol value thus represented the expression level of each protein spot. Expression ratios of spots among cell types were also calculated.

### In-gel Digestion of Differentially Expressed Proteins

Differentially expressed protein spots were excised from the gels and placed in Eppendorf tubes. In-gel digestion was performed according to the procedure published by Havlis *et al.* [26] with minor modifications [23]. Gel plugs were washed twice with double distilled water followed by 50% acetonitrile in 50 mM ammonium bicarbonate and then with pure acetonitrile. Gel plugs were dried in a SpeedVac evaporator (Tokyo Rikakikai, Tokyo, Japan) and subjected to in-gel digestion or stored at -20°C. For digestion, gel plugs were re-swollen with 20 ng/µL trypsin (Promega, Madison, WI, USA) in 25 mM ammonium bicarbonate at 4°C for 30 min, and then at 56°C for 1 h. After digestion, peptide products were recovered by 97.5% acetonitrile and 2.5% trifluoroacetic acid (TFA).

### Identification of Proteins by Matrix-assisted Laser Desorption/ionization- Time of Flight Mass Spectrometry (MALDI-TOF MS) and MALDI-TOF/TOF MS Analysis

Digested peptides were spotted directly onto a 600 µm/384 well AnchorChip™ sample target (Bruker Daltonics, Bremen, Germany), and then added with an equal volume of 1 mg/mL solution of alpha-cyano-hydroxycinnamic acid (CHCA) in 0.1% TFA/50% acetonitrile. The MALDI mass spectra were obtained using a Bruker autoflex TOF mass spectrometer equipped with a 384 sample Scout source (Bruker Daltonics). An external peptide calibration standard containing Angiotensin II ([M+H]^+^1046.54), Angiotensin I ([M+H]^+^1296.68), Substance P ([M+H]^+^1347.74), Bombesin ([M+H]^+^1619.82), ACTH clip 1–17 ([M+H]^+^2093.09), ACTH clip 18–39 ([M+H]^+^2465.20) and Somatostatin 28 ([M+H]^+^3147.47) (Bruker Daltonics) was used to calibrate the instrument, and the spectra were acquired in reflection mode. Peptide masses were searched against a comprehensive nonredundant protein sequence database (NCBInr 20110922 version with 15173690 sequences and 5201003429 residues) or SwissProt database (SwissProt 2012_04 version with 535698 sequences; 190107059 residues) using the Mascot program [26] for protein identification. The search criteria were taxonomy for Mammalia, fixed modification of carbamidomethyl modification, variable modifications of oxidation modification, mass accuracy of 50 to 300 ppm and maximally one missed cleavage site. Positive identification was achieved with minimum 5 peptides matched and with the set mass accuracy and modification when the score matched with significant probability to the protein or mixture of proteins in the database.

The AnchorChip™ target were subjected to acquire MALDI-TOF/TOF spectra using a Bruker autoflex II TOF/TOF mass spectrometer (Bruker Daltonics) equipped with a delayed-extraction ion source. Metastable ions generated by laser-induced decomposition (LID) in the LIFT mode (Bruker Daltonics) were analyzed. The precursor ion and the corresponding fragment ions were selected in a time gate followed by further acceleration in the LIFT cell with 19.0 kV. The fragment ions were accelerated into the second field-free region and were separated in the two-stage gridless reflectron. The reflectron voltage was set at 27.4 kV. Mass spectra were processed using the FlexAnalysis 2.4 software (Bruker Daltonics). The proteins were identified by searching MS/MS spectra against NCBInr database (NCBInr 20110922 version) or SwissProt database (SwissProt 2012_04 version) using BioTools 3.0 software (Bruker Daltonics) in combination with the Mascot program [27]. The search criteria and positive identification were as described in the previous section for MALDI-TOF MS analysis.

### Validation of Protein Expression by Western Blot Analysis

To further validate the differential expression of proteins among cell types, fibroblasts, f-rES, and p-rES cells were collected for Western blotting. Protein extracts were prepared by resuspending cells with 400 µL of sample buffer (0.15 mM NaCl, 5 mM EDTA, 1% Triton-100, 10 mM Tris-HCl, and 5 mM dithiothreitol) containing 0.1 mM protease inhibitor cocktail (Sigma P8340, St. Louis, MO, USA) on ice for 10 min, and then centrifuged at 18,600×g for 10 min at 4°C. The supernatant was stored at -80°C until use.

Prior to electrophoresis, the extracted protein was boiled for 5 min and then loaded on 10% (v/v) SDS-PAGE. The resolved proteins were transferred onto nitrocellulose membranes (Cat. No. HAHY00010, Millipore, Billerica, MA, USA) as described in previous study [8]. After blotting, the membrane was incubated in blocking buffer (5% chick serum in TBST) for 2 h at room temperature and then reacted with antibodies against α-tubulin (Cat. No. T5168, Sigma-Aldrich, 1∶1000), peroxiredoxin 1 (Abcam, Cambridge, MA, USA, 1∶1000), TCP1-α (Cat. No. RabMAB 3179, Epitomics, Burlingame, CA, USA, 1∶1000), HSP60 (Cat. No. SPA-806-D, Stressgen Biotechnologies, Ann Arbor, MI, USA, 1∶1000), HSP70 (Cat. No. SPA-820, Stressgen Biotechnologies, 1∶1000), HSP90 (Cat. No. SPA-830, Stressgen Biotechnologies, 1∶1000), Oct4 (Cat. No. CA, SC8628 Santa Cruz, 1∶1000), Nanog (Cat. No. SC30331 Santa Cruz, 1∶500), and β-actin (Cat. No. 4967, Cell Signaling Technology, Danvers, MA, USA, 1∶1000) at 4°C overnight, respectively. The nitrocellulose membrane was washed with 1× TBST (Tris-buffered saline with Tween-20, blocking solution; 200 mM Tris-HCl, 5 M NaCl, 0.05% Tween-20, pH 7.4) and then reacted with HRP-conjugated secondary antibodies (Cat. No. 28169, Anaspec Inc., Fremont, CA, USA) for 1 h. Proteins on the blots were visualized with a Super-Signal West chemiluminescent substrate kit (Thermo Fisher Scientific Inc., Cambridge, MA, USA) and the intensity of protein signals was determined by using the Image J software.

### Immunocytochemical Staining for Specific Markers

To analyze the expression of specific protein markers, rabbit cells were rinsed with DPBS and then fixed in 4% paraformaldehyde for 2–4 days after culture. After washing with DPBS for 10 min, the cells were permeated in TBST, treated with 0.3% Triton X-100 in DPBS for 30 min, followed by 3 washings in DPBS prior to incubation with blocking solution (DPBS +2% skim milk) for 60 min. The three rabbit cell types were first incubated with primary mouse or human antibodies (anti-α-tubulin, Cat. No. T5168, Sigma-Aldrich; anti-Peroxiredoxin 1, Cat. No. ab41906, Abcam; anti-TCP-1α, Cat. No. RabMAB 3179, Epitomics; anti-HSP60, Cat. No. SPA-806-D, Stressgen Biotechnologies; anti-HSP70, Cat. No. SPA-820, Stressgen Biotechnologies; anti-HSP90, Cat. No. SPA-830, Stressgen Biotechnologies; anti-Oct4, Cat. No. SC8628, Santa Cruz; anti-Nanog, Cat. No. ab115586, Abcam; anti-TRA-1-60, Cat. No. ab16288, Abcam; anti-TRA-1-81, Cat. No.ab16289, Abcam; or anti-SSEA-4, MAB Cat. No. 4304, CHEMICON). All antibodies were diluted in blocking solution performed as previously described [8], [11] and incubated with samples at 4°C overnight after an additional 3 washes (15 min/wash) with 0.05% Tween-20 (Amersham Life Science 20605). After being incubated with the primary antibody, the cells were washed again with DPBST for 3 times and then incubated with the secondary antibodies for 60 min (Peroxiredoxin 1, TCP-1α and SSEA-4, Alexa fluor 488-conjugated goat anti-mouse, Invitrogen, 1∶100; TRA-1-60 and TRA-1-81, Alexa fluor 546-conjugated goat anti-mouse, Invitrogen, 1∶1000; α-tubulin, HSP60, HSP70 and HSP90, 546-conjugated goat anti-rabbit, Invitrogen, 1∶100; Oct4 and Nanog 594-conjugated donkey anti-goat Invitrogen, 1∶1000). Finally, epifluorescent dye DAPI (1 µg/mL) was added to DPBST for nuclear staining, followed by two rinses with DPBST before confocal microscopic examination (LSM 510, Carl Zeiss, Jena, German).

### Bioinfomatic Analysis

The differentially expressed proteins among the three cell types were annotated for their subcellular distribution, biological processes, and molecular function by Gene Ontology (GO) database (http://www.geneontology.org/) and Biocompare (http://www.biocompare.com/). Briefly, unique proteins were uploaded to GO database and searched for their cellular component and molecular function. To simplify the classification, we chose the second or third level of tree browser in molecular function. The biological processes were classified by Biocompare because of their relatively simpler classification.

### Statistical Analysis

The RVol of protein spots on all triplicate 2-DE gels of each cell types were analyzed using the least-squares means method in the general linear model procedure of Statistical Analysis System software [28]. The spots with significant probability (*p<0.05*) and with expression ratio higher than 1.5 were considered significantly different among cell types. The results of Western blot analysis were quantified by using the Image J software and the band density was normalized by using β-actin as an internal standard. The normalized data was analyzed using least-squares means method [28]. The data were presented as means ± standard error and probability at *p<0.05* was considered as significantly different among cell types.

## Results

### Morphology of rES Cells and Comparison of Protein Profiles

**Figure 2:**
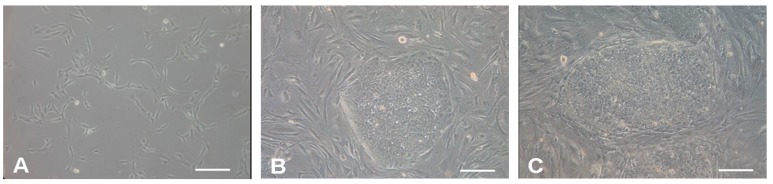
The morphologies of rabbit fibroblast (A), f-rES (B), and p-rES (C) cells grown to log phase. Fertilized-rES cells and p-rES cells were propagated on MEF feeder cells and grown into compact colonies. Scale bar = 100 µm.

**Figure 3:**
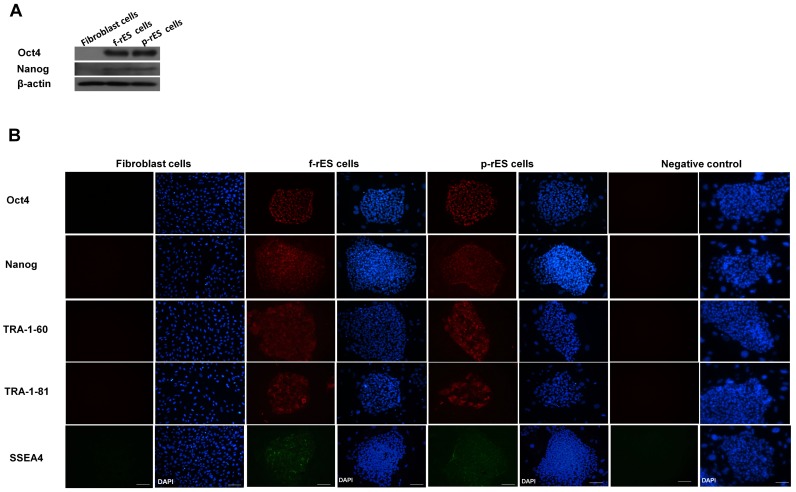
Analyses of expressions of pluripotency related gene in rabbit embryonic stem cells. (A) Western blot analyses of Oct4 and Nanog expressions in rabbit fibroblast, f-rES, and p-rES cells. Note that both f-rES and p-rES cell lines expressed all the pluripotency markers. Beta-actin is served as a loading control. (B) Immunocytochemical analyses of marker expressions of the three cell types (fibroblast, f-rES, and p-rES cells). The rES cell line expressed the markers recognized by antibodies against Oct4, Nanog, TRA-1-60, TRA-1-81, and SSEA-4. The nucleus is labeled by DAPI, and negative control is only stained with secondary antibody without primary antibody. Scale bar = 100 µm.

To identify distinct protein expressions within rES cells of different origins, rabbit fibroblast, f-rES, and p-rES cells were collected and used for 2-DE analyses. Figure 2 shows the morphology of the fibroblast cells (Fig. 2A), f-rES cells (Fig. 2B), and p-rES cells (Fig. 2C) at the log phase of passage 15. Instead of having a 3-D configuration as seen in mES cells, rES cells morphologically resembled hES cells in their flat and compact shape, which could be easily recognized when they were cultured on the feeders. The f-rES and p-rES cells showed positive expressions of Oct4 and Nanog by Western blot analysis (Fig. 3A). We also observed the expressions of SSEA-4, Nanog, Oct4, and the keratin sulfate antigens (TRA-1-60 and TRA-1-81) in the f-rES cells and p-rES cells examined (Fig. 3B) by immunostaining.

**Figure 4:**
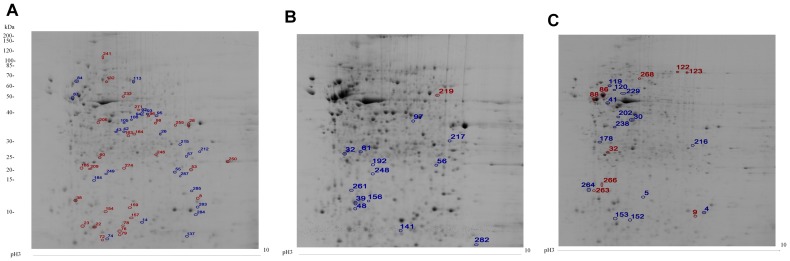
Protein profiling of f-rES (A), fibroblast (B), and p-rES cells (C) grown to log phase. A total of 1,000 µg soluble proteins were TCA precipitated and separated by IEF using 180 mm IPG DryStrips at pH 3–10, followed by 12.5% SDS-PAGE gels. Proteins were visualized by colloidal Coomassie blue staining. Spots with higher expression levels are circled and numbered in red, and those with lower expression levels circled and numbered in blue.

Representative 2-DE protein profiles of each type of cells are shown in Fig. 4. All gels showed a wide distribution of protein spots with pI ranging from 3.0 to 10.0 on 12.5% SDS-PAGE gels, and a mass ranging from 10 to 200 kDa. Of the 284 protein spots quantified among these three cell types, 100 showed distinguishable scale (*p<0.05*) greater than 1.5. To illustrate the differential expressions of proteins among cell types, spots with higher expression levels are circled and numbered in red and those with lower expression levels are in blue (Fig. 4). Fifty-four protein spots with similar expression levels between f-rES and p-rES cells are indicated by circles as shown in Fig. 4A. Among the 54 spots, the expression levels of 29 spots or proteins (in red) represent the proteins with expression levels that are higher in f-rES and p-rES cells than in fibroblasts (Table 1). The other 25 spots (in blue), on the contrary, are those with lower expression levels lower in f-rES and p-rES cells. There were 14 proteins similar in expression levels between f-rES and fibroblast cells (Fig. 4B), and 23 protein spots between p-rES and fibroblast cells (Fig. 4C).

**Table 1:** List of identified Group 1 proteins with similar expression levels in f-rES cells and p-rES cells (*cf.* fibroblast cells).

Spot No.	Protein Identity	fibroblast cells (FB*)	f-rES cells (R4*)	p-rES cells (PA*)	Expression ratio			Cellular component	Function
					R4/FB	PA/FB	R4/PA		
Twenty-nine spots with similar higher expression levels in both f-rES cells and p-rES cells than in fibroblast cells									
163	Annexin A2	0.067±0.040^b^	0.204±0.027^a^	0.246±0.023^a^	3.05	3.67	-1.21	Nucleus	Calcium ion binding
23	ATP synthase, H+ transporting, mitochondrial F1 complex, alpha subunit precursor	0.056±0.047^b^	0.270±0.098^a^	0.234±0.075^a^	4.8	4.15	1.16	Mitochondrion	Transporter activity
60	Beta actin	0.150±0.094^b^	0.416±0.069^a^	0.357±0.056^a^	2.76	2.37	1.16	Cytoskeleton	Structural constituent of cytoskeleton
76	Cofilin-1	0.194±0.032^b^	0.458±0.128^a^	0.614±0.169^a^	2.36	3.16	-1.34	Cytoplasm	Cytoskeletal protein binding
98	Cytoplasmic linker 2	0.122±0.083^b^	0.313±0.106^a^	0.312±0.039^a^	2.6	2.59	1	Cytoplasm	Signal transduction
79	Eukaryotic translation initiation factor 5A-1	0.011±0.010^b^	0.165±0.029^a^	0.133±0.010^a^	14.64	11.79	1.24	Cytoplasm	Translation initiation factor activity
233	Ezrin	0.026±0.012^b^	0.153±0.054^a^	0.132±0.047^a^	5.78	4.99	1.16	Cytoplasm	Cytoskeletal anchoring activity
274	Ferritin light chain 1	0.047±0.015^b^	0.169±0.047^a^	0.146±0.033^a^	3.6	3.12	1.15	Cytoplasm	Storage protein
22	Galectin-1	0.186±0.038^b^	0.899±0.179^a^	1.026±0.152^a^	4.82	5.5	-1.14	Extracellular	Receptor binding
8	Glyceraldehyde 3-phosphate dehydrogenase	0.028±0.008^b^	0.113±0.045^a^	0.127±0.021^a^	4.02	4.53	-1.12	Cytoplasm	Catalytic activity
72	Inositol-1,4,5-triphosphate receptor 1	0.015±0.007^b^	0.129±0.047^a^	0.163±0.054^a^	8.62	10.93	-1.26	Endoplasmic reticulum	Intracellular ligand-gated ion channel activity
132	Lamin-B1	0.049±0.020^b^	0.166±0.030^a^	0.138±0.063^a^	3.37	2.81	1.2	Nucleus	Structural molecule activity
271	Mitochondrial carrier triple repeat 6	0.032±0.001^b^	0.151±0.005^a^	0.205±0.059^a^	4.73	6.42	-1.36	Mitochondrion	Receptor binding
53	Peroxiredoxin 1	0.251±0.052^b^	0.440±0.086^a^	0.502±0.079^a^	1.76	2	-1.14	Cytoplasm	Peroxidase activity
209	Peroxiredoxin 2	0.012±0.005^b^	0.265±0.059^a^	0.305±0.074^a^	22.18	25.51	-1.15	Cytoplasm	Peroxidase activity
250	Peroxiredoxin-1	0.285±0.128^b^	1.097±0.159^a^	0.930±0.414^a^	3.85	3.26	1.18	Cytoplasm	Peroxidase activity
28	PREDICTED: similar to Glyceraldehyde-3-phosphate dehydrogenase	0.549±0.253^b^	1.128±0.106^a^	1.105±0.310^a^	2.06	2.01	1.02	Cytoplasm	Catalytic activity
241	PREDICTED: similar to mitochondrial diablo-like protein isoform 1	0.037±0.008^b^	0.168±0.055^a^	0.234±0.060^a^	4.56	6.35	-1.39		
154	PREDICTED: similar to Tuba1 protein, partial	0.151±0.031^b^	0.261±0.061^a^	0.293±0.063^a^	1.73	1.94	-1.12	Cytoskeleton	Structural constituent of cytoskeleton
164	Protein disulfide-isomerase A3	0.033±0.026^b^	0.174±0.023^a^	0.199±0.020^a^	5.31	6.07	-1.14	Endoplasmic reticulum	Isomerase activity
96	Pyruvate kinase, muscle	0.109±0.067^b^	0.311±0.069^a^	0.304±0.013^a^	2.86	2.79	1.02	Cytoplasm	Catalytic activity
205	S-adenosylmethionine synthase isoform type-2	0.023±0.011^b^	0.098±0.006^a^	0.121±0.048^a^	4.3	5.31	-1.23	Cytoplasm	Catalytic activity
255	Spastin	0.151±0.043^b^	0.286±0.037^a^	0.295±0.060^a^	1.9	1.96	-1.03	Nucleus	Cytoskeletal protein binding
185	T-complex protein 1, isoform CRA_c	0.084±0.034^b^	0.172±0.023^a^	0.189±0.026^a^	2.04	2.24	-1.1	Cytoplasm	Chaperone
246	Triosephosphate isomerase	0.017±0.009^b^	0.173±0.038^a^	0.137±0.019^a^	10.13	8.01	1.27	Cytoplasm	Isomerase activity
38	Tubulin, alpha 4a	0.485±0.116^b^	0.907±0.173^a^	1.025±0.248^a^	1.87	2.12	-1.13	Cytoskeleton	Structural molecule activity
78	unknown	0.009±0.002^b^	0.133±0.036^a^	0.111±0.015^a^	14.39	12	1.2		
157	unknown	0.011±0.010^b^	0.237±0.084^a^	0.240±0.050^a^	22.02	22.33	-1.01		
159	unknown	0.108±0.050^b^	0.298±0.044^a^	0.231±0.037^a^	2.76	2.14	1.29		
Twenty-five spots with similar lower expression levels in both f-rES cells and p-rES cells than in fibroblast cells									
42	PREDICTED: similar to Alpha enolase isoform 3	0.729±0.183^a^	0.202±0.030^b^	0.163±0.041^b^	-3.6	-4.47	1.24	Cytoplasm	Catalytic activity
43	PREDICTED: similar to Alpha-enolase isoform 1	0.530±0.034^a^	0.282±0.103^b^	0.226±0.008^b^	-1.88	-2.34	1.25	Cytoplasm	Catalytic activity
57	Glyceraldehyde-3-phosphate dehydrogenase	0.392±0.036^a^	0.129±0.015^b^	0.165±0.047^b^	-3.03	-2.37	-1.28	Cytoplasm	Catalytic activity
74	Glyceraldehyde-3-phosphate dehydrogenase	0.777±0.213^a^	0.235±0.101^b^	0.213±0.011^b^	-3.3	-3.65	1.11	Cytoplasm	Catalytic activity
92	PREDICTED: similar to Phosphoglycerate kinase 1	1.082±0.273^a^	0.302±0.040^b^	0.273±0.060^b^	-3.58	-3.97	1.11	Cytoplasm	Catalytic activity
93	Pyruvate kinase isozymes M1/M2	0.676±0.041^a^	0.412±0.071^b^	0.291±0.089^b^	-1.64	-2.32	1.41	Cytoplasm	Catalytic activity
95	Pyruvate kinase isozymes M1/M2	1.616±0.116^a^	0.438±0.048^b^	0.375±0.027^b^	-3.69	-4.31	1.17	Cytoplasm	Catalytic activity
105	Pyruvate kinase isozymes M1/M2	0.726±0.219^a^	0.248±0.045^b^	0.202±0.051^b^	-2.93	-3.59	1.23	Cytoplasm	Catalytic activity
106	Pyruvate kinase isozymes M1/M2	0.435±0.065^a^	0.168±0.019^b^	0.143±0.014^b^	-2.59	-3.03	1.17	Cytoplasm	Catalytic activity
184	Glutathione S-transferase	0.215±0.042^a^	0.138±0.023^b^	0.141±0.016^b^	-1.55	-1.52	-1.02	Cytoplasm	Glutathione transferase activity
215	Chain A, Fructose 1,6-Bisphosphate Aldolase	0.293±0.060^a^	0.105±0.020^b^	0.120±0.016^b^	-2.79	-2.45	-1.14	Cytoplasm	Aldolase activity
249	Glutathione transferase	0.190±0.020^a^	0.125±0.011^b^	0.116±0.024^b^	-1.52	-1.63	1.07	Cytoplasm	Glutathione transferase activity
283	Glyceraldehyde-3-phosphate dehydrogenase	0.340±0.115^a^	0.070±0.015^b^	0.061±0.035^b^	-4.88	-5.57	1.14	Cytoplasm	Catalytic activity
285	Glyceraldehyde-3-phosphate dehydrogenase	0.582±0.164^a^	0.138±0.047^b^	0.130±0.009^b^	-4.23	-4.48	1.06	Cytoplasm	Catalytic activity
84	Protein disulfide-isomerase	2.037±0.437^a^	0.933±0.078^b^	1.209±0.524^b^	-2.18	-1.68	-1.3	Endoplasmic reticulum	Isomerase activity
113	Protein disulfide isomerase-associated 3 precursor (predicted)	0.904±0.251^a^	0.491±0.187^b^	0.507±0.048^b^	-1.84	-1.78	-1.03	Endoplasmic reticulum	Isomerase activity
87	Vimentin	4.679±1.243^a^	1.709±0.610^b^	2.511±0.576^b^	-2.74	-1.86	-1.47	Cytoskeleton	Structural constituent of cytoskeleton
14	Myelin expression factor 2	1.297±0.040^a^	0.189±0.010^b^	0.272±0.069^b^	-6.87	-4.77	-1.44	Nucleus	Transcription regulator activity
26	Annexin A2	0.663±0.150^a^	0.232±0.044^b^	0.319±0.028^b^	-2.86	-2.08	-1.38	Nucleus	Calcium ion binding
287	HNRPA1 protein	0.466±0.137^a^	0.021±0.021^b^	0.022±0.006^b^	-21.92	-21.39	-1.03	Nucleoplasm	Nuclear mRNA splicing
55	Phosphatidylethanolamine-binding protein	0.484±0.039^a^	0.324±0.040^b^	0.240±0.055^b^	-1.5	-2.01	1.35	Cytoplasm	Lipid binding
137	Anti-ds-DNA immunoglobulin heavy chain V region	0.285±0.054^a^	0.131±0.044^b^	0.169±0.067^b^	-2.18	-1.69	-1.29		
212	Paraneoplastic antigen like 6A	0.270±0.021^a^	0.089±0.016^b^	0.072±0.011^b^	-3.04	-3.73	1.23		
94	unknown	0.215±0.038^a^	0.081±0.069^b^	0.059±0.030^b^	-2.64	-3.67	1.39		
284	unknown	0.420±0.140^a^	0.161±0.027^b^	0.111±0.022^b^	-2.61	-3.78	1.45		

* The expression levels of protein spots are ratios of the volume of each spot to the overall volume of all the quantified protein spots generated by the Melanie 7 software.

a–b Least squares means with different superscripts differ significantly within the same row (*p<0.05*).

### Identification of the Differentially Expressed Protein Spots

The identities of the differentially expressed protein spots among cell types were resolved by MALDI-TOF and MALDI-TOF/TOF MS. Among the 100 differentially expressed protein spots, 91 were successfully identified in the GenBank deposits which represented 63 different proteins. The detailed information on the identities of these proteins was shown in Table S1.

### Bioinformatic Analyses of the Identified Proteins

**Figure 5:**
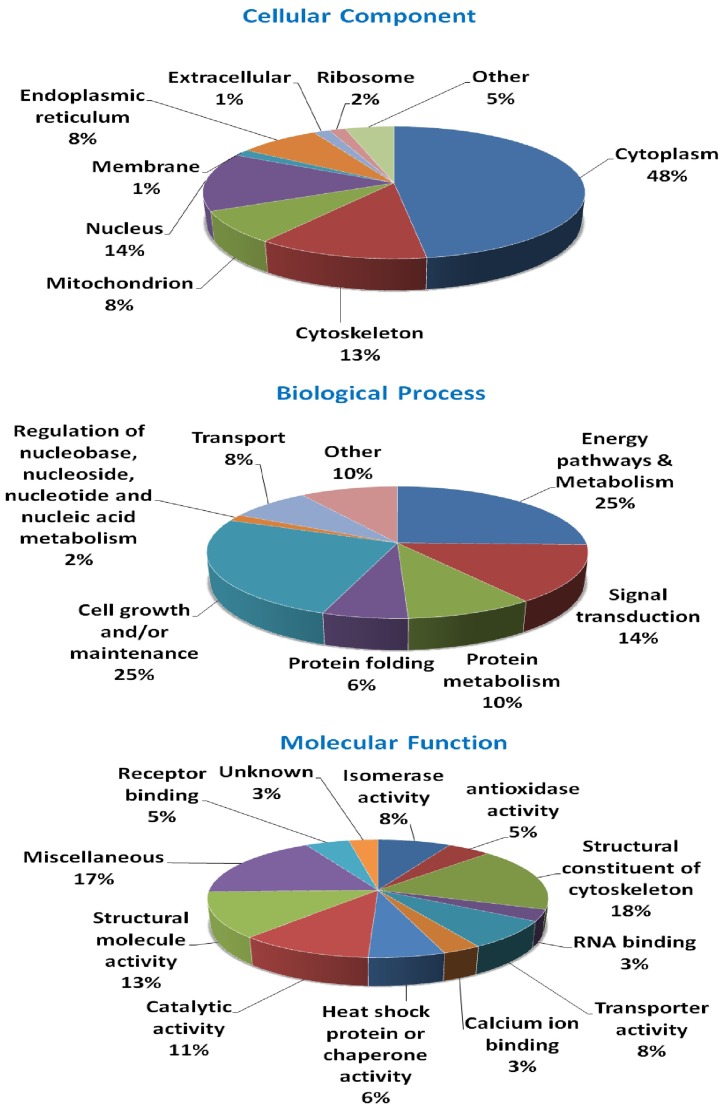
Gene ontology (GO) analysis of differentially expressed proteins among fibroblasts, f-rES, and p-rES cells. The official gene symbols of differentially expressed proteins are used for the GO annotations. Original GO annotations for cellular components, molecular functions, and biological processes are based on the NCBI Entrez Gene database for classification. The percentages represent the total hits divided by the number of annotated proteins within each category.

The gene functions of the 63 differentially expressed proteins among the three cell types are annotated by their GO terms (Fig. 5). When categorized by cellular component, 48% of the annotated proteins are for cytoplasmic, 13% for cytoskeletal, 14% for nuclear, 8% for endoplasmic reticulum, and 8% for mitochondrial proteins. The major biological processes in which these differentially expressed proteins participated are energy metabolisms (25%), cell growth and/or maintenance (25%), signal transduction (14%), and protein metabolisms (10%). In terms of their molecular functions, 18% of the proteins belong to functional or structural constituents of the cytoskeleton, 13% involve in structural molecule activity, 11% catalytic activity, 8% isomerase activity, 8% transporter activity, 6% heat shock proteins (HSP) or chaperones, and 17% with miscellaneous activities.

### Grouping of the Differentially Expressed Proteins among Cell Types

**Figure 6:**
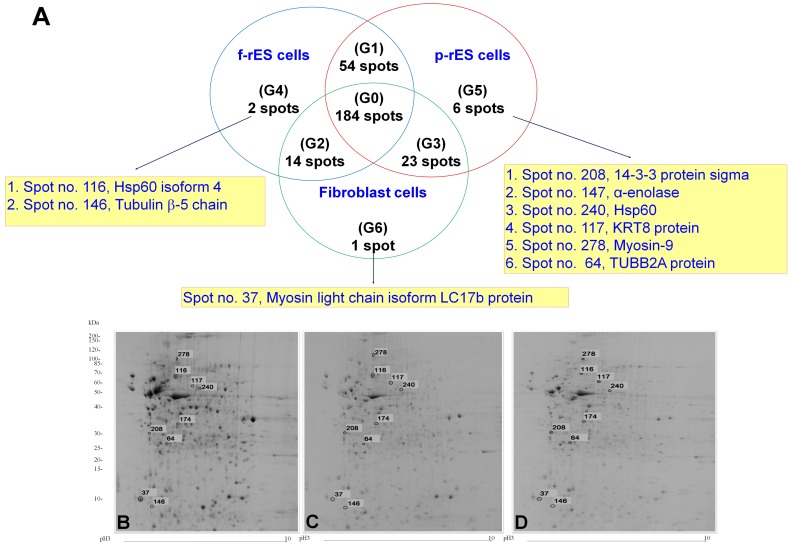
Distributions and classifications of protein expression profiles in rabbit fibroblast, f-rES, and p-rES cells. (A) The 284 quantified protein spots in the three cell types were mandatorily classified into seven groups (G0-G6) based on the similarities or differences in protein expression levels. (B) A representative 2-DE gel (fibroblasts) showing the protein spot (No. 37; G6) exclusively expressed in fibroblast cells. (C) A representative 2-DE gel (f-rES cells) showing the two protein spots (No. 116 and No.146; G4) exclusively expressed in the f-rES cells. (D) A representative 2-DE gel (p-rES cells) shows the six proteins (No. 64, 117, 174, 208, 240, and 278; G5) that are exclusively expressed in p-rES cells. The numbers are the spot numbers in Table 4.

The quantified protein spots are further divided into 7 groups based on their expression levels (Fig. 6). Group 0 (G0) contains 184 protein spots showing a similar expression level among the three cell types. Group 1 (G1) to Group 6 (G6) represent all differentially expressed protein spots (Fig. 6 and Tables 1, 2, 3, 4). Group 1 consists of 54 protein spots with similar expression levels between f-rES and p-rES cells yet excluding those spots in G0 (Fig. 6A), of which 29 spots (Fig. 4A, red circles) had higher expression levels in both f-rES and p-rES cells than in fibroblast cells, and 25 spots (Fig. 4A, blue circles) showed lower expression levels in both f-rES and p-rES cells than in fibroblast cells (Fig. 4A and Table 1).

**Table 2:** List of identified Group 2 proteins with similar expression?levels in f-rES cells and fibroblasts (*cf.* p-rES cells).

Spot No.	Protein Identity	fibroblast cells (FB*)	f-rES cells (R4*)	p-rES cells (PA*)	Expression ratio			Cellular component	Function
					R4/FB	PA/FB	R4/PA		
One spot with similar higher expression in both f-rES cells and fibroblast cells than in p-rES cells									
219	Stress-induced-phosphoprotein 1 (STI1) (Hsc70/Hsp90-organizing protein)	0.233±0.042^a^	0.291±0.025^a^	0.130±0.028^b^	1.25	-1.8	2.25	Nucleus	Receptor signaling complex scaffold activity
Thirteen spots with similar lower expression levels in both f-rES cells and fibroblasts than in p-rES cells									
48	Actin, aortic smooth muscle	0.099±0.011^b^	0.120±0.004^b^	0.430±0.043^a^	1.22	4.36	-3.58	Cytoplasm	Structural constituent of cytoskeleton
192	S-adenosylhomocysteine hydrolase	0.069±0.023^b^	0.084±0.014^b^	0.161±0.033^a^	1.21	2.32	-1.93	Cytoplasm	Hydrolase activity
217	Carbonic anhydrase 2	0.154±0.009^b^	0.170±0.031^b^	0.306±0.052^a^	1.11	1.99	-1.8	Cytoplasm	Catalytic activity
261	VCP protein	0.163±0.006^b^	0.167±0.09^b^	0.353±0.088^a^	1.03	2.16	-2.11	Endoplasmic reticulum	ATPase activity
39	Gamma-actin	0.930±0.245^b^	0.714±0.207^b^	1.530±0.206^a^	-1.3	1.65	-2.14	Cytoskeleton	Structural constituent of cytoskeleton
248	PREDICTED: similar to Cytoplasmic β-actin isoform 2	0.060±0.017^b^	0.063±0.005^b^	0.111±0.017^a^	1.04	1.84	-1.77	Cytoskeleton	Cell motion
33	Annexin A1	0.413±0.064^b^	0.471±0.062^b^	1.543±0.137^a^	1.14	3.73	-3.27	Plasma membrane	Receptor binding
61	Annexin A1	0.191±0.053^b^	0.190±0.041^b^	0.593±0.144^a^	-1.01	3.1	-3.12	Plasma membrane	Receptor binding
56	Mitochondrial F1-Atpase	0.100±0.021^b^	0.150±0.025^b^	0.264±0.032^a^	1.5	2.64	-1.76	Mitochondrion	Transporter activity
86	Vimentin	5.481±0.756^a^	3.411±0.914^b^	4.364±1.134^a^	-1.61	-1.26	-1.28	Cytoskeleton	Structural constituent of cytoskeleton
282	Ribosomal protein S2	0.328±0.067^b^	0.440±0.127^b^	0.768±0.181^a^	1.34	2.34	-1.74	Ribosome	Ribosomal subunit
97	Unknown	0.133±0.040^b^	0.150±0.037^b^	0.320±0.110^a^	1.12	2.4	-2.14		
141	Unknown	0.131±0.044^b^	0.140±0.002^b^	0.211±0.026^a^	1.06	1.61	-1.51		
156	Unknown	0.026±0.027^b^	0.029±0.015^b^	0.169±0.071^a^	1.13	6.49	-5.75		

* The protein expression level is ratio of the volume of each spot to the overall volumes of all the quantified protein spots generated by the Melanie 7 software.

a–b Least squares means with different superscripts differ within the same row (*p<0.05*).

**Table 3:** List of identified Group 3 proteins with similar expression levels in p–rES cells and fibroblasts (*cf*. f-rES cells).

Spot No.	Protein Identity	fibroblast cells (FB^*^)	f-rES cells (R4^*^)	p-rES cells (PA^*^)	Expression ratio				Cellular component	Function
					R4/FB		PA/FB	R4/PA		
Eight spots with similarly higher expression levels in p-rES cells and fibroblasts than in f-rES cells										
9	Glyceraldehyde 3-phosphate dehydrogenase	0.200±0.007^a^	0.082±0.015^b^	0.151±0.051^a^	-2.42	-1.32		-1.83	Cytoplasm	Catalytic activity
32	Tubulin, beta 5	0.711±0.106^a^	0.340±0.045^b^	0.650±0.166^a^	-2.09	-1.09		-1.91	Cytoskeleton	Structural constituent of cytoskeleton
88	Vimentin	1.492±0.035^a^	0.623±0.128^b^	1.322±0.305^a^	-2.39	-1.13		-2.12	Cytoskeleton	Structural constituent of cytoskeleton
122	Caldesmon 1	0.391±0.091^a^	0.123±0.063^b^	0.496±0.105^a^	-3.18	1.27		-4.04	Cytoplasm	Cytoskeletal protein binding
123	Caldesmon 1	0.443±0.096^a^	0.182±0.095^b^	0.597±0.106^a^	-2.44	1.35		-3.29	Cytoplasm	Cytoskeletal protein binding
263	unnamed protein product	0.110±0.022^a^	0.047±0.011^b^	0.121±0.019^a^	-2.34	1.1		-2.56	?	?
266	Myosin regulatory light chain, LC20	0.283±0.050^a^	0.182±0.045^b^	0.318±0.053^a^	-1.55	1.12		-1.75	Cytoskeleton	Structural molecule activity
268	unknown	0.188±0.017^a^	0.099±0.007^b^	0.150±0.040^a^	-1.89	-1.25		-1.51	?	?
Fourteen spots with similarly lower expression levels in p-rES cells and fibroblasts than in f-rES cells										
178	14-3-3 Gamma In Complex With A Phosphoserine Peptide	0.147±0.034^b^	0.266±0.031^a^	0.131±0.043^b^	1.82	-1.12		2.03	Cytoplasm	Receptor signaling complex scaffold activity
216	Carbonic anhydrase II	0.075±0.024^b^	0.197±0.053^a^	0.084±0.018^b^	2.63	1.13		2.34	Cytoplasm	Energy & Metabolism
152	Cellular retinoic acid-binding protein 1	0.121±0.050^b^	0.372±0.106^a^	0.167±0.052^b^	3.08	1.39		2.23	Cytoplasm	Transporter activity
4	Cyclophilin 18	2.145±0.566^b^	3.449±0.877^a^	1.644±0.306^b^	1.61	-1.3		2.1	Cytoplasm	Catalytic activity
5	Cyclophilin 18	0.089±0.014^b^	0.188±0.032^a^	0.069±0.036^b^	2.12	-1.28		2.71	Cytoplasm	Catalytic activity
153	Glutathione S-transferase mu 2	0.189±0.046^b^	0.416±0.100^a^	0.223±0.048^b^	2.2	1.18		1.86	Cytoplasm	Energy & Metabolism
41	Heat shock 70 kDa protein 8	0.743±0.115^b^	1.870±0.280^a^	0.909±0.067^b^	2.52	1.23		2.06	Cytoplasm	Chaperone activity
202	Heat shock protein HSP 90-beta	0.231±0.082^b^	0.477±0.041^a^	0.183±0.017^b^	2.07	-1.26		2.6	Melanosome	Chaperone activity
120	Mitochondrial ATP synthase, H+ transporting F1 complex beta subunit	0.239±0.114^b^	0.536±0.138^a^	0.259±0.136^b^	2.24	1.08		2.07	Mitochondrion	Transporter activity
229	PREDICTED: similar to Heterogeneous nuclear ribonucleoprotein F (hnRNP F) isoform 1	0.349±0.040^b^	0.734±0.094^a^	0.445±0.056^b^	2.1	1.28		1.65	Nucleoplasm	Nuclear mRNA splicing
264	TUBB protein	0.733±0.165^b^	1.319±0.119^a^	0.840±0.235^b^	1.8	1.15		1.57	Cytoskeleton	Structural constituent of cytoskeleton
30	TUBB2A protein	0.913±0.340^b^	2.079±0.166^a^	0.967±0.225^b^	2.28	1.06		2.15	Cytoplasm	Structural constituent of cytoskeleton
238	TUBB2A protein	0.175±0.042^b^	0.375±0.061^a^	0.184±0.015^b^	2.14	1.05		2.04	Cytoskeleton	Structural constituent of cytoskeleton
119	Tubulin beta-5 chain	0.436±0.141^b^	0.939±0.132^a^	0.519±0.187^b^	2.16	1.19		1.81	Plasma membrane	Structural constituent of cytoskeleton

The protein expression level is the ratio of the volume of each spot to the overall volume of all the quantified protein spots generated by the Melanie 7 software.

a–b Least-squares means with different superscripts differ within the same row (*p<0.05*).

**Table 4:** Identified Groups 4, 5, and 6 proteins with differential expression levels in f-rES, p-rES, and fibroblast cells, repectively.

Spot No.	Protein Identity	fibroblast cells (FB*)	f-rES cells (R4*)	p-rES cells (PA*)	Expression ratio				Cellular component		Function		
					R4/FB		PA/FB	R4/PA					
Group 4: Two spots with the highest expression level in f-rES cells than in p-rES cells and fibroblast cells													
116	PREDICTED: similar to Heat shock protein 60 isoform 4	0.525±0.130^b^	0.859±0.201^a^	0.245±0.019^c^	1.63	-2.15		3.51		Mitochondrion	Heat shock protein activity		
146	Tubulin beta-5 chain	0.069±0.014^c^	0.170±0.021^a^	0.115±0.014^b^	2.46	1.66		1.48		Plasma membrane	Structural constituent of cytoskeleton		
Group 5: Six spots with the highest expression level in p-rES cells than in f-rES cells and fibroblast cells													
64	TUBB2A protein	0.135±0.064^c^	0.018±0.002^b^	0.342±0.060^a^	-7.55		2.54	-19.17	Cytoplasm		Structural constituent of cytoskeleton		
117	KRT8 protein	0.035±0.016^c^	0.455±0.098^b^	0.779±0.141^a^	12.92		22.11	-1.71	Cytoplasm		Structural molecule activity		
174	Alpha-enolase	0.096±0.027^c^	0.151±0.006^b^	0.231±0.014^a^	1.58		2.41	-1.53	Cytoplasm		Catalytic activity		
208	14-3-3 protein sigma	0.056±0.008^c^	0.274±0.022^b^	0.644±0.073^a^	4.85		11.4	-2.35	Cytoplasm		Receptor signaling complex scaffold activity		
278	Myosin-9	0.018±0.008^c^	0.065±0.029^b^	0.128±0.015^a^	3.57		7.05	-1.98	Nucleus		Microfilament motor activity		
240	Heat shock protein 60	0.042±0.008^c^	0.083±0.018^b^	0.138±0.029^a^	2		3.31	-1.66	Mitochondrion		Heat shock protein activity		
Group 6: One spot with the highest expression level in fibroblast cells than in f-rES cells and p-rES cells													
37	Myosin light chain isoform LC17b	1.047±0.064^a^	0.213±0.023^c^	0.613±0.012^b^	-4.91	-1.71		-2.88		Cytoplasm		Structural molecule activity	

* The protein expression level is the ratio of the volume of each spot to the overall volumes of all the quantified protein spots generated by the Melanie 7 software.

a–c Least-squares means with different superscripts differ within the same row (*p<0.05*).

Group 2 represents 14 protein spots with similar expression levels between f-rES and fibroblast cells (Fig. 4B and Table 2); one spot has a higher expression level (in red) and 13 spots are with lower levels (in blue) in both f-rES and fibroblast cells than in those of p-rES cells. Group 3 contains 23 protein spots with similar expression levels in p-rES and fibroblast cells (Fig. 4C, Table 3); nine protein spots have higher expressions and 14 spots are lower expressed in p-rES and fibroblast cells than that in f-rES cells.

The two protein spots exclusively expressed in f-rES cells are grouped as G4 (Fig. 6A, Table 4). By molecular weight, they are similar to heat shock protein 60 isoform 4 (spot No. 116) and tubulin β-5 chain (spot No. 146). These spots are indicated in 2-DE protein gel of f-rES cells (Fig. 6C, Table 4). Six protein spots with specifically up-regulated expression in p-rES cells are grouped as G5, and they are TUBB2A protein (spot No. 64), KRT8 protein (spot No. 117), α-enolase (spot No. 174), 14-3-3 protein sigma (spot No. 208), HSP60 (spot No. 240), and myosin-9 (spot No. 278) (Fig. 6A, Table 4). These spots are indicated in the 2-DE gel of p-rES cells (Fig. 6D, Table 4). Only myosin light chain isoform LC 17b protein (spot No. 37) was exclusively expressed in fibroblast cells (G6), and is indicated in the 2-DE protein gel of fibroblast cells (Fig. 6B, Table 4).

### Validation and Confirmation of the Differentially Expressed Proteins by Western Blot Analysis and Immunocytochemistry

**Figure 7:**
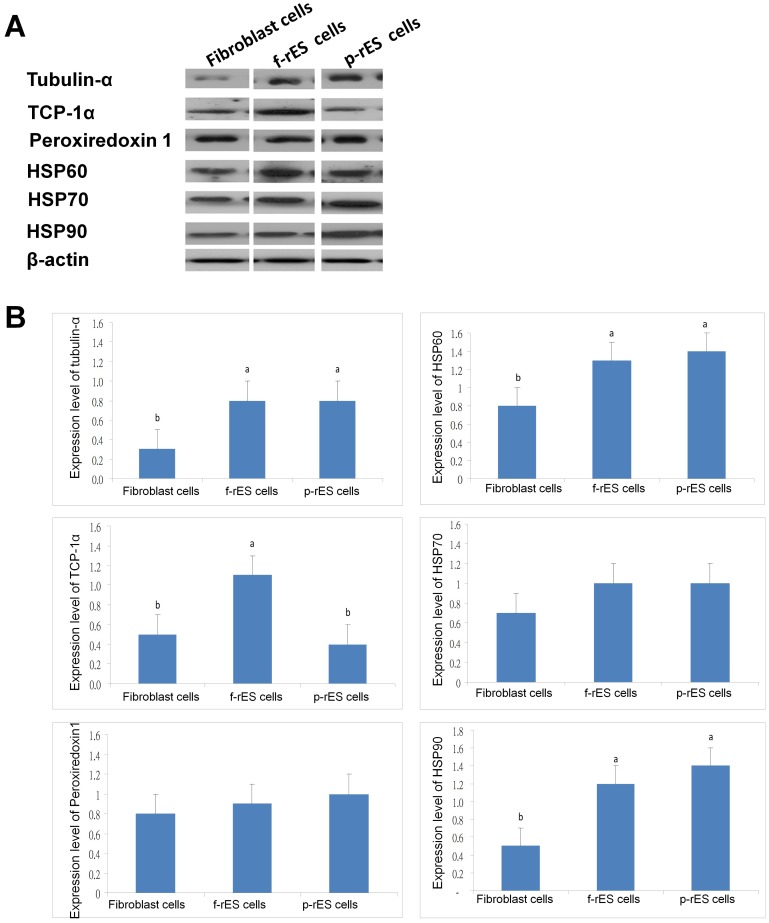
Western blot analysis of total protein extracts from fibroblast, f-rES and p-rES cells. (A) The proteins of interest were analyzed using 50 µg of protein extracts from fibroblast, f-rES and p-rES cells for SDS-PAGE followed by Western blotting. Membranes were hybridized with antibodies against α-tubulin, peroxiredoxin 1, TCP-1α, HSP60, HSP70, and HSP90, using β-actin as a loading control. The protein bands in the blots were quantified by using an Image J software, and the quantitative data is shown in (B). Three replicates were performed in each cell type, and bars without the same alphabetic letters differ (*p<0.05*).

**Figure 8:**
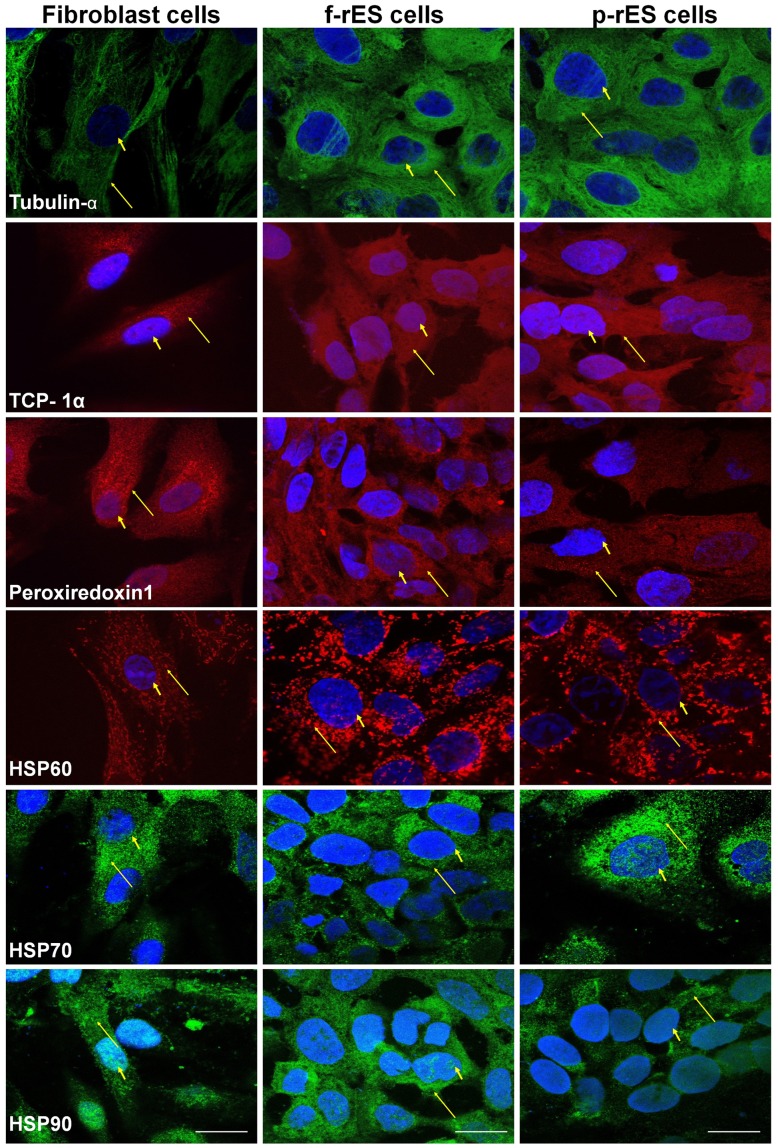
Immunocytochemical confirmation and subcellular localization of the selected proteins in fibroblast, f-rES, and p-rES cells. The cells were hybridized with primary antibodies against α-tubulin, peroxiredoxin 1, TCP-1α, HSP60, HSP70, and HSP90 and then incubated with the secondary antibodies (Peroxiredoxin 1 and TCP-1α, Alexa fluor 488-conjugated goat anti-mouse; α-tubulin, HSP60, HSP70 and HSP90, 546-conjugated goat anti-rabbit). The epifluorescent dye DAPI (1 µg/mL) was used for nuclear staining. The images were observed by confocal microscopy. Yellow arrows indicate protein locations in the cell, and arrow heads indicate the nucleus by DAPI staining. Bar = 20 µm.

To corroborate the proteomic analysis, the relative expression of selected proteins differentially expressed was also compared among cell types by Western blot (Fig. 7) and immunocytochemistry (Fig. 8) analyses. Alpha-tubulin, peroxiredoxin 1, and TCP-1α protein were all expressed in the fibroblast, f-rES, and p-rES cells (Fig. 7A). Quantitative analysis showed that the α-tubulin had a higher expression in both f-rES and p-rES cells than in fibroblast cells (Fig. 7B). The α-tubulin was mostly localized in several cellular compartments of ES cells (Fig. 8). Similar to α-tubulin, TCP-1α showed a higher expression level (*p<0.05*) in f-rES cells than in fibroblasts by Western blotting (Fig. 7). Furthermore, TCP-1α was localized in the cytoplasm and cell cortex (Fig. 8). These results indicate that TCP-1α is important for the development and function of cytoskeletal components of rabbit cells. Peroxiredoxin 1 was expressed in all three cell types (Fig. 7A, B), as confirmed by the Western blot analysis and immunofluorescence staining (Fig. 8).

The differential expression of major HSPs was also observed by 2-DE. Western blot analysis confirmed that the expression levels of HSP60 and HSP90 in both f-rES and p-rES cells are higher than those in fibroblasts (*p<0.05*; Fig. 7). Immunofluorescent assay also confirmed that HSP60 and HSP90 are localized at the cytoplasm of rabbit cells (Fig. 8). Although immunocytochemical analysis indicated that HSP70 was localized in the cytoplasm of all three cell types (Fig. 8), result of Western blot analysis failed to confirm significant differences of this protein among f-rES, p-rES cells and fibroblasts (*p*>0.05).

### Analysis of Imprinted Genes in rES Cells by RT-PCR

**Figure 9:**
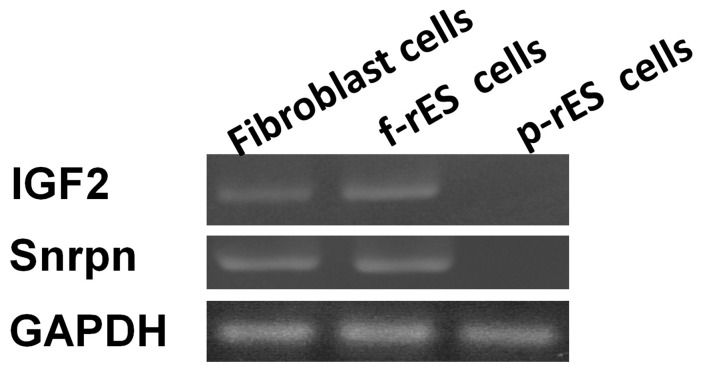
The imprinted gene expressions in fibroblast, f-rES, and p-rES cells. Rabbit fibroblasts and f-rES cell line (R4) all expressed *Igf2* and *Snrpn*, but not the p-rES cells line (A2).

The expression of representative imprinted genes in rES cells were also determined (Fig. 9). The f-rES cells (R4) and fibroblasts expressed the paternally imprinted (*Snrpn* and *Igf2)* genes, but not in p-rES cell line (A2). This finding provided evidence for that our p-rES cell lines originated exclusively from the parthenogenetically activated oocytes without the involvement of paternal genome, and that the *Snrpn* and *Igf2* genes are also imprinted in the rabbit.

## Discussion

Rabbits are phylogenetically closer to humans than rodents and have been extensively used as model animals for the study of human physiology and diseases. In the present study, both f-rES and p-rES cells expressed the same pluripotency markers (Fig. 3), but they still differed in the expression profile of imprinted (*Snrpn* and *Igf2*) genes [4],[12] (Fig. 9). While the gene and protein expressions underlying the pluripotency of f-rES and p-rES cells are largely unknown, this study investigated the protein profiles of these cell lines by a proteomics approach using rabbit fibroblast cells as the control. Among these cells, 100 out of 284 protein spots (∼35%) differed in the expression levels, of which 91 protein spots representing 63 distinct proteins were identified. The proteins with known identities were mainly located in the cytoplasmic compartment and involved in energy and metabolic pathways. Some proteins were expressed exclusively in a specific cell type, indicating a specific nature or physiologic function of each cell type. For instance, at least six proteins including TUBB2A protein, KRT8 protein, α-enolase, 14-3-3 protein sigma, HSP60, and myosin-9 were expressed at significantly higher levels only in p-rES cells (Fig. 6). Two molecules (HSP60 isoform 4 and tubulin β-5 chain) and myosin light chain isoform LC17b protein showed significantly higher expression levels only in f-rES and fibroblasts, respectively.

### Protein Profiles of p-rES Cells and Identification of the Differentially Expressed Proteins

Parthenote-derived ES cells are generated from embryos consisting of only the female genome. It has been reported that the absence of paternal alleles in parthenotes resulted in the lack of paternally imprinted gene expressions, which may have restricted the development of parthenotes [29]–[30]. We have already confirmed that there were no paternally imprinted genes such as *Snrpn* and *Igf2* expressed in the p-rES cells [12]. In this study, 2-DE analysis showed that both upregulated and downregulated proteins were observed in p-rES cells. These proteins may be associated with the expression of maternally imprinted genes. Upregulated proteins of p-rES cells are presented in two groups (Group 2, Table 2; Group 5, Table 4). Group 2 consists of actin, S-adenosylhomocysteine hydrolase, carbonic anhydrase 2, VCP protein, ?-actin, cytoplasmic β-actin isoform 2, annexin A1, mitochondrial F1-ATPase, and ribosomal protein S2 (Fig. 4B and Table 2). Group 5 consists of TUBB2A protein, KRT8 protein, α-enolase, 14-3-3 protein Sigma, HSP60, and myosin-9 (Fig 6A, 6D and Table 4). On the other hand, two downregulated proteins of p-rES cells were stress-induced-phosphoprotein 1 (STI1) at Group 2 (spot No. 219; Fig. 4B and Table 2) and heat shock protein 60 isoform 4 (Group 4, spot No. 116; Fig. 6D and Table 4). These proteins are likely, at least in part, associated with the expression of the maternally imprinted genes in p-rES cells, which require more studies to identify their physiological functions.

### Bioinformatic Analysis of the Differentially Expressed Proteins

The 63 differentially expressed proteins among the three cell types were subjected to GO annotation. Most of these proteins involve in the biological processes of energy pathways, metabolisms and cell growth, and/or maintenance (Fig. 5). Proteins involved in energy pathways and metabolic processes partake in catalytic and antioxidant activities, such as α-enolase, glutathione gyclophilin 18, glyceraldehyde 3-phosphate dehydrogenase, and pyruvate kinase. Some differentially expressed proteins, including tubulin β-5 chain, TUBB2A, KRT8, myosin-9, and myosin light chain isoform LC17b, associated with the cytoskeletal infrastructure are involved in cell growth or maintenance, structural constituent of the cytoskeleton, and their functional molecule activity [31]–[34]. Fourteen percent of the differentially expressed proteins are related to signal transduction (Fig. 5) which may potentially play roles in maintaining the stemness and differentiation capacity of rES cells.

### Putative Roles of Differentially Expressed Proteins in rES Cells

Representative proteins highly expressed in rES cells were selected for further validation by Western blot and immunocytochemical analyses. The expression patterns of TCP-1α, α-tubulin, HSP60, and HSP90 were the most prominent proteins in f-rES and p-rES cells revealed by both Western blot and immunocytochemical assays (Figs. 7 and 8). Of these, cytoskeletal proteins (e.g. actins and tubulins) are among the most abundantly expressed proteins in eukaryotic cells [35]. Tubulins are the major components of the filamentous structure of cellular microtubules with α-tubulin being the most common one [36]. The microtubule plays many crucial roles in intracellular transport, cell morphology, polarity, signaling, and division of the cell [37], which also make it a target for the study of cancer therapy [35]–[38]. In this study, we found that α-tubulin or tubulin-β was upregulated in both f-rES and p-rES cells (Figs. 6, 7, 8), strongly suggesting that ES cells are one of the actively proliferating cell types compared to the terminally differentiated fibroblasts. Moreover, previous studies have also shown that α-tubulins in mES cells are downregulated along with vimentin, one of the intermediate filaments, during differentiation into neuronal cell lineages [16], [39].

The TCP-1 complex is an oligomeric particle found in the eukaryotic cytosol consisting of four or five related polypeptides of a similar size (55–60 kDa) [40]. *In vitro* studies suggested that TCP-1 complex is a chaperonin in the eukaryotic cytosol participating in the correct folding of newly translated α- and β-tubulins and refolding of urea-denatured tubulins and actins in rabbit reticulocyte lysates [41]–[44]. It is also functionally linked to cell growth and its expression decreases concomitantly with the growth arrest during differentiation [45]. Most interestingly, it has been reported that TCP-1 is related to the growth and survival during pig embryo development, and it is more drastically upregulated in pig parthenogenetic embryos than in fertilized embryos [46]. In this study, TCP-1α was found expressed in all the three cell types (Fig. 8) with higher expression levels in rES cells detected by 2-DE (Table 1), particularly highly expressed in f-rES cells detected by Western blotting (Fig. 7). Although the exact cause for the slightly inconsistency between the two analyses is not clear, we infer that TCP-1α may play active roles in cell proliferation and/or cytoskeletal protein folding at least in f-rES cells. Further study is required to determine the precise role of TCP-1α in maintaining the stemness and undifferentiation of rES cells.

Peroxiredoxins are a family of small (22–27 kDa) nonseleno peroxidases in mammals with six isoforms (Peroxiredoxin 1–6) widely distributed in human cells including reproductive organs [47]–[48]. They function to serve as reactive oxygen species (ROS) detoxifiers in order to provide cytoprotection from internal and external environmental stresses by eliminating hydrogen peroxide from cells [49]–[51]. Peroxiredoxins 1 and 2 were highly expressed in ovary and testis [47], [52]. In the female, peroredoxin 1 gene expresses in 3-day-old follicles and increases its expression in 21-day-old during folliculogenesis in the rat [53]. In addition to being found in human endometrium and cervix-vagina fluid [54] and in the epithelium and the endometrial stroma of the uterus, peroredoxins 2 has been reported to be present in the mouse ooplasm of primary follicles, secondary follicles or premature follicles [55]. It is possible that peroxiredoxins also function similarly in rabbits. The results of this study showed that peroxiredoxins 1 and 2 were upregulated in rES cells (Table 1; spot No. 53, 209, and 250). The high peroxiredoxin levels in rabbit ES cells may be associated with their active proliferation capacity and at least partially linked to their future differentiation competency. However, Western blot analysis and immunocytochemistry failed to confirm a similar upregulation in rES cells. Reasons for this discrepancy are unclear, but it could be due to the stressful *in vitro* culture conditions, which might have leveled up its expressions in all three cell types, or the co-existence of other isoforms detected in this study which warrant further investigation.

Heat shock proteins are ubiquitously expressed, and are transcriptionally regulated [56] under physiological and stressful conditions such as elevated temperatures, oxygen tension and chemical insults [57]–[59]. The best characterized roles of HSPs are the involvement of chaperone-mediated protein folding [60]. In the mouse, downregulation of HSPs and the co-chaperones (HSP70 and HSP60) in mES cell lines upon differentiation was observed [61]–[63]. Proteomic analysis of this study showed that two protein spots (spot No. 240) of HSP60 were highly expressed in rES rather than in fibroblast cells (Table 4). Western blot and immunocytochemical analyses both confirmed the 2-DE results (Figs. 7B and 8). HSP60 has been known to bind directly with the *Oct4* and *Nanog* genes which directly regulate *Oct4* and other stemness genes involving the differentiation of adipose tissue-derived stem cells (hATSC) in humans [64]–[65]. The finding in this study confirmed the observations in previous study that undifferentiated cells expressed more HSPs which might be attributable to their active protein synthesis or maintaining pluripotency-related cellular activities, compared to the terminally differentiated cells in the relatively quiescent state.

The expression of HSP90 was only upregulated in f-rES cell lines and showed similar lower levels in fibroblasts and p-rES cells based on our 2-DE analyses (Table 3). It has been reported that the chaperoning activity of HSP90 depends on its ability to hydrolyze ATP and its potential to form stable complexes with HSP70 and HSP70/HSP90/organizing protein (HOP) in fertilized mouse embryo-derived ES cells [64]–[65]. The HOP is a 60 kDa co-chaperone that binds and regulates the activity of chaperones [66], [67]. Using RNAi to knockdown HOP in mES cell lines caused 68% depletion of STAT3 mRNAs, downregulated soluble phosphotyrosine-STAT3 levels, and leading to an extranuclear accumulation of STAT3, which ultimately reduced *Nanog* mRNA levels and lost the ability to form embryoid bodies [68]. These studies confirmed the previous work showing that HSP90 interacted with the JAK/STAT3 signaling molecules in somatic cells [69]. The HSP90 was reported to complex with STAT3 in human embryonic kidney carcinoma cells [70]. Western blot analysis did not completely confirm the differential expression of HSP90 observed in 2-DE analysis in different cell types (Fig. 7). The discrepancy was unclear. In general, HSP90 was upregulated in both f-rES cells and p-rES cells, but remained low in fibroblasts. It might be due to the passage number of rES cells used for analysis, or the commercially available HSP90 antibodies recognized different isoforms of HSP90. However, it has been reported that HSP90 was upregulated in both f-rES and p-rES cells, where the involvement of the proteostatic maintenance of onco-proteins [71] or stemness [64] were demonstrated. Recently, it was also reported that LIF promotes the interaction of HSP90 with STAT3 for maintenance of self-renewal in mES cells [64]. These works provide strong supportive evidence to our previous [8], [12] and current proteomics findings that LIF addition was essential for maintaining self-renewal and the stemness of both f-rES and p-rES cells.

**Figure 10:**
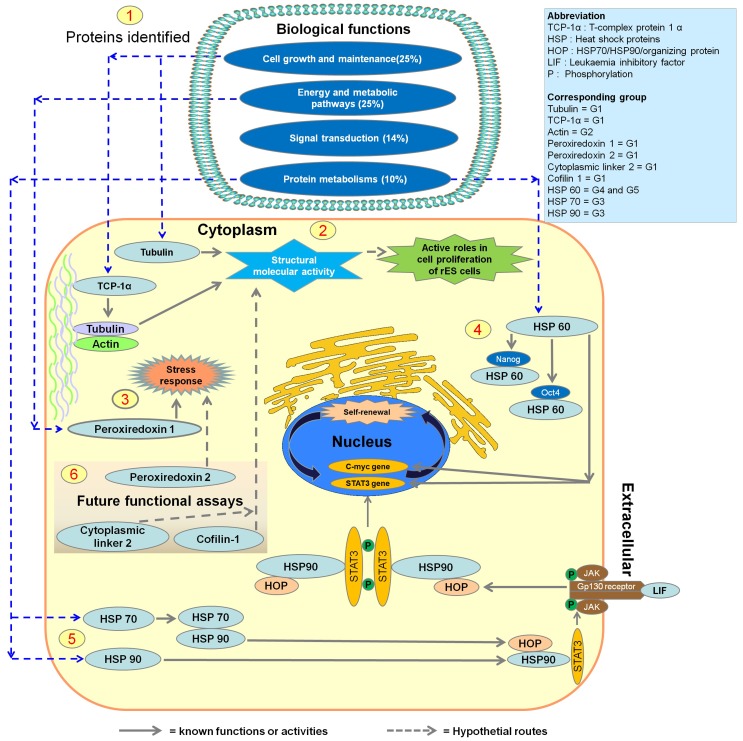
An integrative diagram for presumptive cellular roles played by the representative molecules that distinguish the three rabbit cell types. (1) In the present study, proteins identified are mainly involved in biological functions of energy and metabolic pathways, cell growth and maintenance, signal transduction and protein metabolism. We hypothesize that the selected six proteins are associated with structural constituents of the cytoskeleton, catalytic activity, molecular chaperones, and heat shock proteins (HSP60, HSP70, and HSP90) which were all found expressed in many cell types. (2) Alpha-tubulin, and TCP-1α were expressed with higher expression levels in both rES than in fibroblasts. It is likely that these proteins may play an active role in cell proliferation and/or as regulative proteins for the cytoskeleton rES cells. (3) Peroxiredoxin 1 functions to serve as reactive oxygen species (ROS) detoxifiers to protect cytoplasm from internal and external environmental stresses. (4) HSP60 may bind some pluripotency proteins (e.g. Oct4 and Nanog) or genes (*c-myc* and *Stat3*) that are associated with stress tolerance and/or maintenance of stemness in ES cells. (5) On the other hand, HSP90 hydrolyzes ATP and forms a stable complex with HSP70 and HSP70/HSP90/organizing protein (HOP), which is a 60 kDa co-chaperone that binds and regulates the activity of chaperones related to the LIF/STAT signaling pathway. It has also been reported that HOP down-regulates soluble phosphotyrosine, leading to an extranuclear accumulation of STAT3 in LIF/STAT pathway, which ultimately reduced Nanog mRNA levels and lost the ability of ES cells to form embryoid bodies. (6) Some other molecules, such as peroxiredoxin 2, cytoplasmic linker 2 and cofilin-1, are actively expressed in rabbit ES cells. Peroxiredoxin 2 may play an antioxidant protective role in ES cells. Cytoplasmic linker 2 has been reported as a microtubule plus-end tracking protein that maintains microtubule dynamics by nucleation of non-centrosomal microtubules originated from the trans-Golgi network (TGN). Cofilin-1 is a membrane receptor for estrogen to regulate the mobility of ES cells. The corresponding groups (Table 1, 2, 3, 4) of the mentioned proteins were also listed.

Based on the results from this study and the related reports, we propose an integrative model for the presumptive roles played by the representative molecules that distinguish these three rabbit cell types (Fig. 10). In this model, the α-tubulin and TCP-1α proteins may play an active role in cell proliferation and/or as regulative proteins for the cytoskeleton in rES cells [41]–[44]. Peroxiredoxin 1 functions to serve as a reactive oxygen species (ROS) scavenger or detoxifier to protect cytoplasm from internal and external environmental stresses [49]–[51]. It has been suggested that HSP60 is associated with the stemness of ES cells, in which it may bind some pluripotency proteins (e.g. Oct4 and Nanog) or genes (*c-myc* and *Stat3*) that are related to stress tolerance and/or maintenance of ES cell pluripotency [64]–[65]. In addition, HSP90 hydrolyzes ATP and forms a stable complex with HSP70 and HOP which binds and regulates the activity of chaperones related to the LIF/STAT signaling pathway [64]. In this context, some other key molecules, such as peroxiredoxin 2, cytoplasmic linker 2 and cofilin-1, which are all expressed in rabbit ES cells, may have actively taken part in maintaining normal cellular functions of rES cells.

## Conclusion

To the best of our knowledge, this study demonstrated, for the first time, that the differential proteomic profiles among rabbit ES cells and somatic fibroblasts. The results showed that the characterized proteins for cellular components, biological processes, and molecular functions are those mostly involved in the cytoplasmic functions, energy metabolisms, and structural constituents of the cytoskeleton. The results also provided substantial evidence for the fundamental differences between rabbit somatic cells and ES cells. Furthermore, some key proteins active in rabbit ES cells could be useful cues to re-design the derivation and culture systems for generation of the ground state or germline competent rabbit ES cell lines.

## Supporting Information

Table S1 Protein identities of the differentially expressed proteins in fibroblast, f-rES and p-rES cells. ^&^ Spot numbers are the numbers labeled in Fig. 4. # Numbers in the column are the results from the MALDI-MS PMF analysis, i.e. the number of assigned peptides and percent sequence-coverage (in brackets); ns: no significant match. na: not analyzed.
(DOC)
