# Supplementary material for: Proteomic Profiling of Rabbit Embryonic Stem Cells Derived from Parthenotes and Fertilized Embryos
Source: PLoS One. 2013 Jul 4;8(7):e67772. doi: 10.1371/journal.pone.0067772 (PMC3701598; doi:10.1371/journal.pone.0067772)
Supplement: Table S1 — Protein identities of the differentially expressed proteins in fibroblast, f-rES and p-rES cells. & Spot numbers are the numbers labeled in Fig. 4. # Numbers in the column are the results from the MALDI-MS PMF analysis, i.e. the number of assigned peptides and percent sequence-coverage (in brackets); ns: no significant match. na: not analyzed. (DOC) [file pone.0067772.s001.doc]

Table S1. Protein identities of the differentially expressed proteins in fibroblast, f-rES and p-rES cells.

| Spot no.^&^ | Protein Identity | Gene Symbol | GeneBank Accession | Source species | MALDI-MS PMF^#^ | MALDI-MS-MS | Theoretical Mr/pI |
| --- | --- | --- | --- | --- | --- | --- | --- |
| 4 | Cyclophilin 18 | PPIA | gi|126722924 | Oryctolagus cuniculus | 15/52(86) | 3 (68, 91, 127) | 18054/6.96 |
| 5 | Cyclophilin 18 | PPIA | gi|126722924 | Oryctolagus cuniculus | 9/33(64) | 2 (55, 99) | 18054/6.96 |
| 8 | Glyceraldehyde 3-phosphate dehydrogenase | GAPDH | gi|72496432 | Oryctolagus cuniculus | ns | 2 (65, 90) | 17002/9.46 |
| 9 | Glyceraldehyde 3-phosphate dehydrogenase | GAPDH | gi|126723533 | Oryctolagus cuniculus | ns | 1 (57) | 36025/8.51 |
| 14 | Myelin expression factor 2 | MYEF2 | gi|32425486 | Homo sapiens | 8/25 (47) | na | 22600/9.36 |
| 22 | Galectin-1 | LGALS1 | gi|193442 | Mus musculus | ns | 2 (48, 47) | 15138/5.32 |
| 23 | ATP synthase, H+ transporting, mitochondrial F1 complex, α-subunit precursor | ATP5A1 | gi|4757810 | Homo sapiens | ns | 2 (50, 81) | 59828/9.16 |
| 26 | Annexin A2 | ANXA2 | gi|27807289 | Bos taurus | 12/45(44) | ns | 38873/6.92 |
| 28 | PREDICTED: similar to Glyceraldehyde-3-phosphate dehydrogenase | GAPDH | gi|149268472 | Mus musculus | 10/48(37) | 1 (61) | 40284/8.09 |
| 30 | TUBB2A protein | TUBB2A | gi|109939814 | Bos taurus | 18/64(52) | 2 (54, 95) | 36980/4.47 |
| 32 | Tubulin, β 5 | TUBB5 | gi|7106439 | Mus musculus | 18/53(39) | 1 (63) | 50095/4.78 |
| 33 | Annexin A1 | ANXA1 | gi|1703316 | Oryctolagus cuniculus | 12/60(38) | 4 (48, 77, 64, 41) | 38996/6.28 |
| 37 | Myosin light chain isoform LC17b | MYL6 | gi|253578 | Sus scrofa | 13/44(76) | 1 (61) | 16991/4.46 |
| 38 | Tubulin, alpha 4a | TUBA4A | gi|77735360 | Bos taurus | 11/55(24) | 3 (45, 65, 69) | 50510/4.96 |
| 39 | Gamma-actin | ACTG1 | gi|6478616 | Cavia porcellus | 7/49(73) | 1 (55) | 11023/5.27 |
| 41 | Heat shock 70 kDa protein 8 | HSPA8 | gi|77415383 | Mus musculus | 16/63(30) | 1(49) | 62288/ 5.30 |
| 42 | PREDICTED: similar to α-enolase isoform 3 | ENO1 | gi|73956718 | Canis familiaris | 11/54(27) | 1 (44) | 42807/ 8.03 |
| 43 | PREDICTED: similar to α-enolase isoform 1 | ENO1 | gi|149695415 | Equus caballus | 14/54(35) | ns | 47509/6.37 |
| 48 | Actin, aortic smooth muscle | ACTA2 | ACTA_BOVIN | Bos taurus | ns | 1 (31) | 42381/5.23 |
| 53 | Peroxiredoxin 1 | PRDX1 | gi|55824562 | Macaca fascicularis | 9/45(57) | 1 (62) | 21081/7.10 |
| 55 | Phosphatidylethanolamine-binding protein | PEBP (PBP) | gi|126723727 | Oryctolagus cuniculus | 10/35(59) | ns | 21095/6.59 |
| 56 | Mitochondrial F1-ATPase | ATP5A1 | gi|1943080 | Bos taurus | 11/28 (21) | ns | 55313/8.27 |
| 57 | Glyceraldehyde-3-phosphate dehydrogenase | GAPDH | gi|1177672 | Sus scrofa | ns | 1 (45) | 4140/5.45 |
| 60 | Beta actin | ACTB | gi|12240086 | Oryctolagus cuniculus | ns | 1 (47) | 15814/4.74 |
| 61 | Annexin A1 | ANXA1 | gi|1703316 | Oryctolagus cuniculus | 9/31 (34) | 2 (71, 48) | 38996/6.28 |
| 64 | TUBB2A protein | TUBB2A | gi|109939814 | Bos taurus | 9/39 (30) | ns | 36980/ 4.47 |
| 72 | Inositol-1,4,5-triphosphate receptor 1 | ITPR1 | ITPR1_RAT | Rattus norvegicus | 14/24 (7) | ns | 316486/5.71 |
| 74 | Glyceraldehyde-3-phosphate dehydrogenase | GAPDH | gi|72496432 | Oryctolagus cuniculus | ns | 2 (69, 86) | 17002/9.46 |
| 76 | Cofilin-1 | CFL1 | COF1_MOUSE | Mus musculus | 5/33 (40) | na | 18776/8.22 |
| 78 | unknown |  |  |  | ns | ns |  |
| 79 | Eukaryotic translation initiation factor 5A-1 | EIF5A | gi|124231 | Oryctolagus cuniculus | ns | 1 (50) | 16976/5.08 |
| 84 | Protein disulfide-isomerase | P4HB | gi|129730 | Oryctolagus cuniculus | 19/56 (42) | 3 (83, 55, 85) | 57172/4.77 |
| 86 | Vimentin | VIM | gi|31982755 | Mus musculus | 43/85 (78) | 2 (74, 69) | 53712/5.06 |
| 87 | Vimentin | VIM | gi|2078001 | Mus musculus | 30/60 (70) | 1 (76) | 51590/4.96 |
| 88 | Vimentin | VIM | gi|860908 | Cricetulus griseus | 35/83 (82) | 2 (60, 96) | 44611/4.75 |
| 92 | PREDICTED: similar to Phosphoglycerate kinase 1 | PGK1 | gi|149744840 | Equus caballus | 13/57 (38) | 3 (82, 56, 50) | 48409/7.97 |
| 93 | Pyruvate kinase isozymes M1/M2 | PKM2 | KPYM_RABIT | Oryctolagus cuniculus | 20/77 (42) | ns | 58524/7.62 |
| 94 | unknown |  |  |  | ns | ns |  |
| 95 | Pyruvate kinase isozymes M1/M2 | PKM2 | KPYM_RABIT | Oryctolagus cuniculus | 16/64 (40) | 2 (51, 80) | 58524/7.62 |
| 96 | Pyruvate kinase, muscle | PKM2 | gi|31981562 | Mus musculus | 16/42 (34) | ns | 58378/7.18 |
| 97 | unknown |  |  |  | ns | ns |  |
| 98 | cytoplasmic linker 2 | CYLN2 | gi|149063106 | Rattus norvegicus | 12/33 (14) | ns | 116260/6.11 |
| 105 | Pyruvate kinase isozymes M1/M2 | PKM2 | KPYM_RABIT | Oryctolagus cuniculus | 18/49 (36) | na | 58524/7.62 |
| 106 | Pyruvate kinase isozymes M1/M2 | PKM2 | KPYM_RABIT | Oryctolagus cuniculus | 16/38 (34) | ns | 58524/7.62 |
| 113 | Protein disulfide isomerase-associated 3 precursor (predicted) | PDIA3 | gi|217030873 | Oryctolagus cuniculus | 29/60 (56) | 2 (45, 44) | 56671/5.98 |
| 116 | PREDICTED: similar to Heat shock protein 60 isoform 4 | HSPD1 | gi|74005074 | Canis familiaris | 12/40 (28) | 1 (63) | 57835/5.47 |
| 117 | KRT8 protein | KRT8 | gi|62913980 | Homo sapiens | 14/43 (29) | 1(58) | 41083/4.94 |
| 119 | Tubulin beta-5 chain | TUBB5 | TBB5_BOVIN | Bos taurus | 23/52 (40) | 1 (56) | 50095/4.78 |
| 120 | Mitochondrial ATP synthase, H+ transporting F1 complex β subunit | ATP5B | gi|89574025 | Oryctolagus cuniculus | 28/56 (71) | 3 (80, 56, 46) | 45550/5.21 |
| 122 | caldesmon 1 | CALD1 | gi|21704156 | Mus musculus | 7/18 (22) | ns | 60531/6.97 |
| 123 | caldesmon 1 | CALD1 | gi|21704156 | Mus musculus | 21/46 (46) | na | 60531/6.97 |
| 132 | Lamin-B1 | LMNB1 | gi|50415798 | Homo sapiens | 13/38 (40) | ns | 38289/5.37 |
| 137 | Anti-ds-DNA immunoglobulin heavy chain V region | | gi|736418 | Mus musculus | 4/29 (47) | ns | 13539/9.48 |
| 141 | unknown |  |  |  | ns | ns |  |
| 146 | Tubulin β-5 chain | TUBB5 | TBB5_BOVIN | Bos taurus | 11/49 (29) | 1 (90) | 50095/4.78 |
| 152 | Cellular retinoic acid-binding protein 1 | CRABP1 | RABP1_BOVIN | Bos taurus | 13/44 (73) | 1 (65) | 15753/5.30 |
| 153 | Glutathione S-transferase mu 2 | GSTM2 | gi|126722906 | Oryctolagus cuniculus | ns | 1 (71) | 25572/6.32 |
| 154 | PREDICTED: similar to Tuba1 protein, partial | TUBA1 | gi|149615606 | Ornithorhynchus anatinus | 6/34 (58) | 2 (47, 68) | 13921/5.25 |
| 156 | unknown |  |  |  | ns | ns |  |
| 157 | unknown |  |  |  | ns | na |  |
| 159 | unknown |  |  |  | ns | na |  |
| 163 | Annexin A2 | ANXA2 | gi|27807289 | Bos taurus | 15/62 (38) | ns | 38873/6.92 |
| 164 | Protein disulfide-isomerase A3 | PDIA3 | gi|112293264 | Mus musculus | 11/70 (19) | ns | 57099/5.88 |
| 174 | Alpha-enolase | ENO1 | gi|34784434 | Mus musculus | 51/11 (42) | ns | 40099/5.86 |
| 178 | 14-3-3 Gamma In Complex With A Phosphoserine Peptide | YWHAG | gi|82407948 | Homo sapiens | 8/37 (34) | ns | 28325/4.80 |
| 184 | Glutathione S-transferase | GST | gi|253581 | Bos taurus | ns | 1 (64) | 2491/9.00 |
| 185 | T-complex protein 1, isoform CRA_c | CCT7 | gi|148670090 | Mus musculus | 10/54 (56) | 1 (62) | 17189/4.71 |
| 192 | S-adenosylhomocysteine hydrolase | AHCY | gi|262263372 | Mus musculus | 10/36 (23) | ns | 48170/6.08 |
| 202 | Heat shock protein HSP 90-β | HSP90AB1 | gi|194378142 | Homo sapiens | 13/50 (21) | 1 (45) | 82518/4.98 |
| 205 | S-adenosylmethionine synthase isoform type-2 | | METK2_HUMAN | Homo sapiens | ns | 2 (56, 71) | 43975/6.02 |
| 208 | 14-3-3 protein sigma |  | 1433S_HUMAN | Homo sapiens | 6/37 (21) | ns | 27871/4.68 |
| 209 | Peroxiredoxin 2 | PRDX2 | gi|148747558 | Mus musculus | 10/48 (50) | ns | 21936/5.20 |
| 212 | Glyceraldehyde-3-phosphate dehydrogenase | GAPDH | G3PT_MOUSE | Mus musculus | ns | 2 (43, 58) | 48367/8.14 |
| 214 | Triosephosphate isomerase | TPI1 | gi|136066 | Oryctolagus cuniculus | 10/25 (53) | ns | 26894/7.10 |
| 215 | Chain A, Fructose 1,6-Bisphosphate Aldolase | ALDOA | gi|2781027 | Oryctolagus cuniculus | 14/39 (53) | ns | 39647/8.30 |
| 216 | Carbonic anhydrase II | CA2 | gi|118582299 | Oryctolagus cuniculus | 10/35 (50) | 1 (46) | 29596/6.95 |
| 217 | Carbonic anhydrase II | CA2 | gi|118582299 | Oryctolagus cuniculus | 16/34 (56) | ns | 29596/6.95 |
| 219 | Stress-induced-phosphoprotein 1 (STI1) (Hsc70/Hsp90-organizing protein) | STIP1 | gi|73983760 | Canis lupus familiaris | 11/28 (22) | ns | 63148/6.35 |
| 229 | PREDICTED: similar to Heterogeneous nuclear ribonucleoprotein F (hnRNP F) isoform 1 | HNRNPF | gi|57107167 | Canis familiaris | 16/48 (42) | 1 (49) | 45948/5.32 |
| 233 | Ezrin | VIL2 | gi|126723108 | Oryctolagus cuniculus | 13/49 (25) | ns | 69291/6.09 |
| 238 | TUBB2A protein | TUBB2A | gi|109939814 | Bos taurus | 19/53 (53) | ns | 36980/4.47 |
| 240 | Heat shock protein 60 | HSPD1 | gi|76779273 | Mus musculus | 17/31 (36) | ns | 59559/8.09 |
| 241 | PREDICTED: similar to mitochondrial diablo-like protein isoform 1 | DIABLO | gi|149720695 | Equus caballus | 7/25 (33) | na | 26851/5.78 |
| 246 | Triosephosphate isomerase | TPI1 | gi|1864018 | Mus musculus | 59/11 (75) | 2 (50, 64) | 22720/5.62 |
| 248 | PREDICTED: similar to cytoplasmic β-actin isoform 2 | ACTB | gi|109119055 | Macaca mulatta | 8/41 (39) | 2 (55, 93) | 28478/5.20 |
| 249 | Glutathione transferase | GST | gi|29135329 | Bos taurus | ns | 1 (61) | 23826/6.89 |
| 250 | Peroxiredoxin-1 | PRDX1 | gi|66773956 | Bos taurus | 18/49 (70) | 2 (102, 147) | 22423/8.59 |
| 255 | Spastin | SPG4 | gi|47523346 | Sus scrofa | 10/43 (27) | na | 58515/9.57 |
| 261 | VCP protein | VCP | gi|112818458 | Homo sapiens | 13/43 (20) | 1 (44) | 52769/4.88 |
| 263 | unnamed protein product |  | gi|90086105 | Macaca fascicularis | 7/37 (62) | ns | 19790/4.71 |
| 264 | TUBB protein | TUBB | gi|38511503 | Homo sapiens | 14/59 (54) | 1 (70) | 14465/4.23 |
| 266 | Myosin regulatory light chain, LC20 | MYL9 | gi|264748 | Sus scrofa | 11/38 (57) | ns | 19740/4.80 |
| 268 | unknown |  |  |  | ns | ns |  |
| 271 | Mitochondrial carrier triple repeat 6 | MCART6 | gi|123121892 | Mus musculus | 6/38 (26) | ns | 26073/9.26 |
| 274 | Ferritin light chain 1 | FTL1 | FRIL1_MOUSE | Mus musculus | 9/45 (55) | 1 (39) | 20847/5.66 |
| 278 | Myosin-9 | MYH9 | gi|32484326 | Mus musculus | 8/25 (29) | ns | 43682/5.83 |
| 282 | Ribosomal protein S2 | RPS2 | gi|2293579 | Bos taurus | ns | 1 (60) | 30796/10.31 |
| 283 | Glyceraldehyde-3-phosphate dehydrogenase | GAPDH | gi|89573917 | Oryctolagus cuniculus | 7/26 (31) | ns | 25180/8.87 |
| 284 | unknown |  |  |  | ns | ns |  |
| 285 | Glyceraldehyde-3-phosphate dehydrogenase | GAPDH | gi|89573917 | Oryctolagus cuniculus | ns | 2 (41, 67) | 25180/8.87 |
| 287 | HNRPA1 protein | HNRNPA1 | gi|157834095 | Homo sapiens | 15/36 (77) | ns | 20898/7.26 |
